# From structure prediction to ligand–target recognition: expanding the reach of AlphaFold in natural product research

**DOI:** 10.1007/s13659-026-00654-8

**Published:** 2026-11-03

**Authors:** Guangrui Xu, Haifeng Hu, Weihui Zhao, Zhengxi Liu, Ting Wang, Rui Liu, Song Liu

**Affiliations:** 1 Institute of Pharmaceutical Process, Hubei Province Key Laboratory of Occupational Hazard Identification and Control, School of Medicine, Wuhan University of Science and Technology, Wuhan, 430065 China; 2 China State Institute of Pharmaceutical Industry Co Ltd, Shanghai, 200232 China

**Keywords:** AlphaFold, Natural products, Structure prediction, Peptides, Protein–protein interaction, Co-folding, Molecular glues

## Abstract

Natural products constitute a structurally diverse reservoir of bioactive molecules and remain an important source of lead compounds for drug discovery. However, the limited availability of structural information on pharmacological targets and biomolecular interactions continues to hinder mechanism elucidation and structure-guided optimization, particularly for complex natural products such as bioactive peptides, proteinaceous natural products, and natural molecular glues. Recent advances in AlphaFold have transformed structural biology by enabling high-confidence prediction of protein structures and biomolecular complexes, providing unprecedented opportunities for structure-guided natural product research. Beyond conventional structure-based virtual screening, emerging co-folding strategies have demonstrated considerable potential for improving early enrichment in natural product screening and for elucidating the binding mechanisms of complex natural products with their targets. This review summarizes recent advances in AlphaFold-assisted natural product research, including target structure prediction, hit and lead discovery, co-folding-enabled interaction analysis, mechanistic investigation of peptide- and protein-derived natural products, and the emerging potential of AlphaFold for natural molecular glue discovery. We further discuss current limitations and future directions in structure prediction and interaction modeling. Overall, AlphaFold is reshaping natural product research from lead discovery to mechanistic characterization, with particular promise for advancing macromolecular natural products and molecular glue-mediated therapeutics.

**Graphical abstract:**

**Figure:**
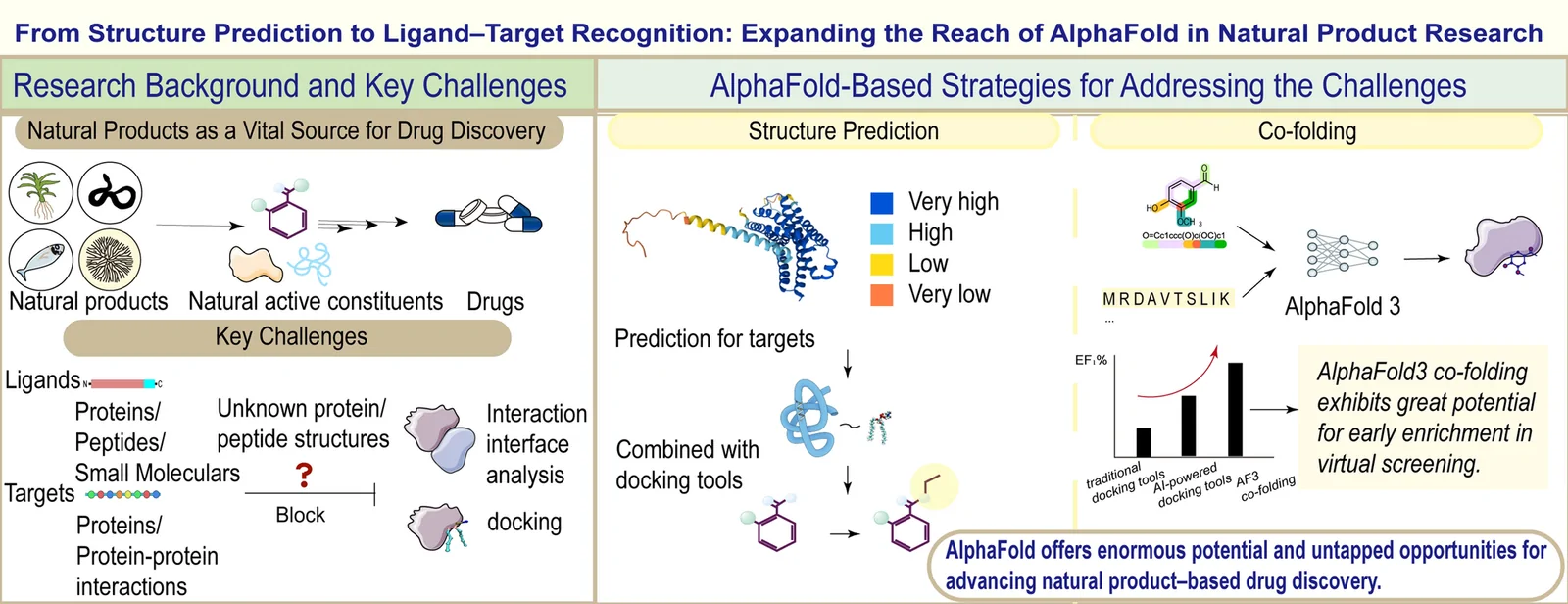

## Highlights

- AlphaFold (AF) modeling is integrated into natural product (NP) drug discovery.
- AF-based target structural prediction promotes NP-derived hit-to-lead optimization.
- Advantages of AF3-based co-folding in early enrichment of screening are outlined.
- AF-driven macro-NP structural prediction and interaction with targets are reviewed.
- Challenges and future developments of AF in NP drug discovery are discussed.

## Introduction

Natural products (NPs) are widely distributed in plants, animals, microorganisms, and marine organisms, forming a structurally diverse reservoir of bioactive constituents [1, 2]. Their unique chemical diversity and broad biological activities have made them an important source of pharmaceuticals, nutraceuticals, dietary supplements, and cosmetics [3–8]. In fact, more than half of FDA-approved drugs originate from NPs [9], underscoring their pivotal role in disease prevention and treatment, as well as their substantial translational and commercial potential [10].

Owing to the vast chemical diversity of NPs, considerable efforts have been devoted to identifying bioactive small molecules through structure-based virtual screening (SBVS), which provides a rapid, high-throughput, and cost-effective strategy for target identification, hit discovery, and mechanism investigation [11–13]. However, the effectiveness of SBVS is often constrained by the limited availability of experimental target structures, as protein structure determination and target validation remain labor-intensive, costly, and technically challenging [14, 15]. Beyond small-molecule NPs acting on monomeric proteins, growing attention has recently been directed toward proteinaceous and peptide NPs, as well as natural compounds that modulate protein–protein interactions (PPIs). However, the conformational flexibility of peptide natural products (PNPs) complicates accurate structural modeling. Another limitation is the insufficient characterization of target–ligand interactions (e.g., protein–small molecule, protein–peptide, and protein–protein complexes), which hampers molecular mechanism studies. Finally, docking performance is compromised at protein–protein or protein–peptide interfaces, which are typically flat and lack well-defined binding pockets, resulting in low enrichment efficiency due to expanded conformational search space and inadequate scoring functions [16–20].

**Fig. 1:**
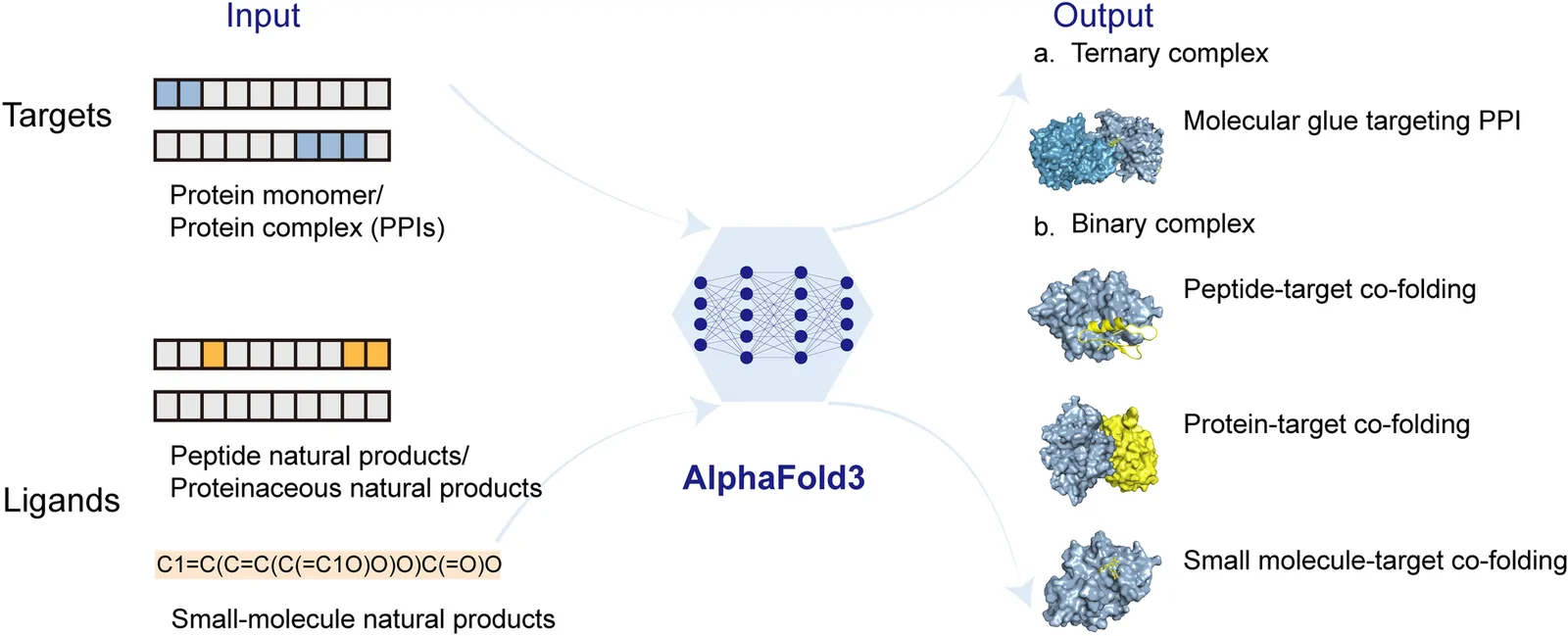
Schematic illustration of the AlphaFold3 (AF3) co-folding strategy with natural products (NPs). The diagram illustrates the input and output framework of AF3 co-folding, where target proteins and NP ligands are jointly modeled to predict the 3D structures of their complexes

AlphaFold3 (AF3), a deep learning-based model developed by Google DeepMind, represents a transformative breakthrough for tackling these challenges. Based on a diffusion-based architecture, AF3 predicts structures for diverse molecular entities, including proteins, nucleic acids, small molecules, ions, and modified residues, with accuracy surpassing that of many conventional approaches [21–24]. AlphaFold (AF) enables the accurate prediction of protein–protein and protein–peptide interactions, thereby extending NP research beyond classical small-molecule systems to include PPI-targeting and macromolecular NPs [25–27]. Meanwhile, AF-derived structures can also serve as high-confidence templates for virtual screening and lead optimization [28–30]. Concurrently, AF-based co-folding (Fig. 1) serves as an innovative complementary strategy, allowing for de novo prediction of bound complexes directly from molecular inputs. These advances have opened new opportunities for studying molecular glues (MGs), whose mechanisms rely on stabilizing PPIs and are often difficult to capture using conventional docking-based approaches [31, 32].

Although numerous reviews have highlighted the role of AF in structure prediction and virtual screening, a systematic perspective on how AF expands the scope of NP research remains lacking. To address these gaps, this review provides a structured overview of AF-based approaches in the research of NPs, highlighting their expanding role in macromolecular NPs and co-folding-based drug discovery.

## Evolution of AlphaFold and the principles and advantages of AlphaFold3

### Iterative development

**Fig. 2:**
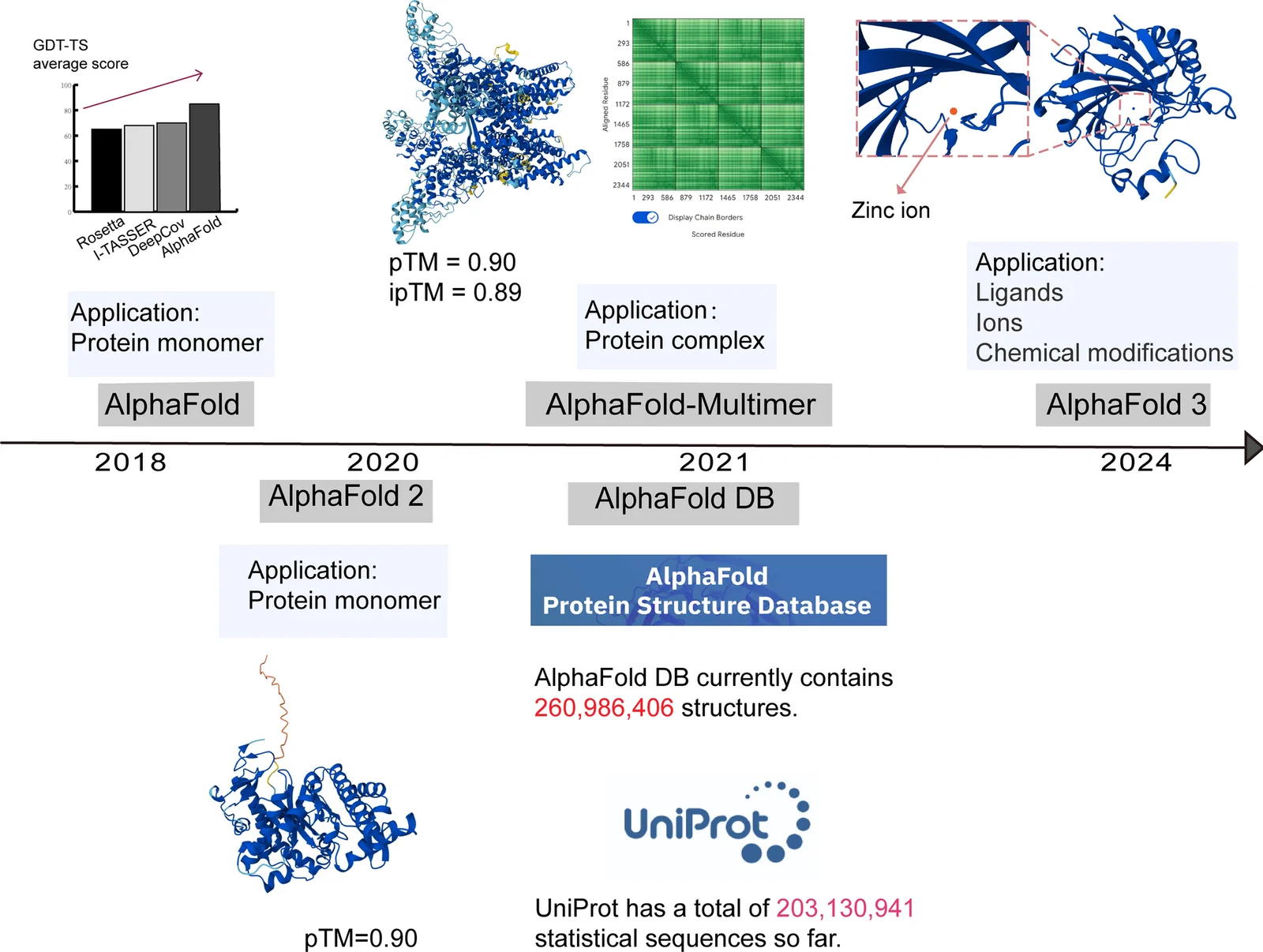
Development of the AF series and expansion of its predictive capabilities. The timeline highlights key milestones from monomeric protein prediction to protein complex and ligand-containing complex modeling across successive AF series

AF first gained widespread recognition at the CASP competition in 2018, achieving accuracy comparable to the state-of-the-art template-based methods without relying on templates. Since then, AF has undergone iterative development, advancing from the initial version [33] to AlphaFold2 (AF2) [34], the AlphaFold Protein Structure Database (AlphaFold DB) [35], AlphaFold Multimer (AFM) [36], and ultimately to AF3 [21] (Fig. 2). Across these iterations, both predictive accuracy and the scope of addressable biological systems have been substantially improved. The comparative features of these versions, including model architecture, predictive capabilities, and application scenarios, are summarized in Table 1.

**Table 1:** Comparison of the AF-based tools

Tools	Model architectures				Prediction objects		Application scenarios	References
	MSA	Diffusion model	Pairformer	Evoformer	Monomer	Complex		
AF3	✓	✓	✓	×	✓	✓	Drug–target interaction analysis; study on cell function and disease mechanism; basic research on molecular structure	[21]
AF2	✓	×	×	✓	✓	×	Basic studies on protein structure	[34]
AFM	Based on AF2				✓	✓	Protein complex structure prediction	[36]
AlphaFold DB	Database				✓	×	Structural models for drug development based on protein complex targets	[35]

✓: present/supported; ×: absent/not supported

### Network architecture

**Fig. 3:**
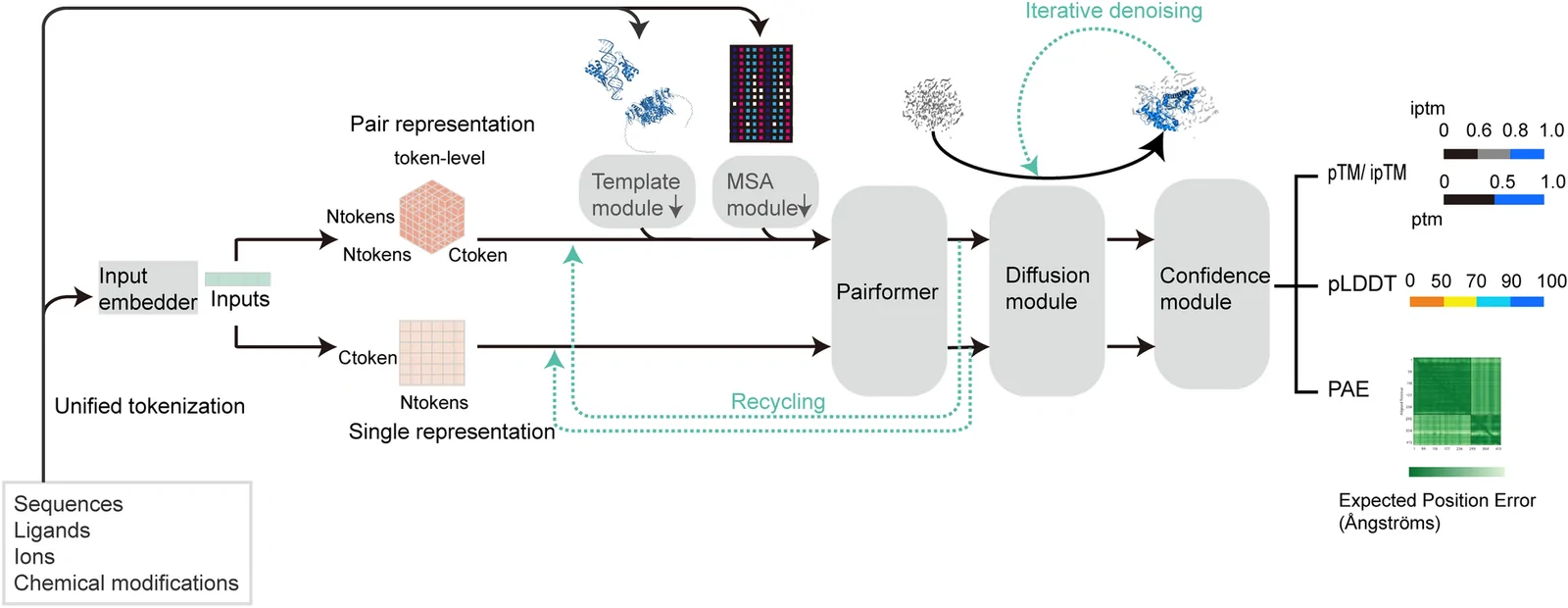
Schematic overview of the innovative structural prediction framework of AF3. Compared with AF2, AF3 particularly reduces dependence on multiple sequence alignment (MSA) and template information

Compared to AF2, DeepMind introduced significant architectural innovations in AF3. As illustrated in Fig. 3, these improvements can be broadly categorized into four major components [21]:

Firstly, AF3 adopts a hierarchical and multi-scale transformer architecture that simultaneously models local structural details and global spatial organization, endowing a strong generalization capability across diverse protein systems [37].

Secondly, the Pairformer module deviates from the heavy reliance on multiple sequence alignment (MSA) by operating on both single and pair representations [36, 38]. Triangle update integrates pairwise residue information; triangle self-attention enhances modeling of critical residue-residue interactions; transition layers update single representations; and single attention with pair bias captures residue-level dependencies by incorporating pairwise structural information [21, 34].

Thirdly, AF3 introduces a diffusion-based generative module, which frames structure generation as a progressive forward-noising and reverse-denoising process [39, 40]. During the forward process, protein structures are gradually perturbed into random noise, establishing a reversible probabilistic mapping. During the reverse process, deep neural networks learn the noise distribution and reconstruct backbone conformations and torsion angles from stochastic inputs through progressive denoising toward experimentally realistic conformations [41, 42].

Finally, AF3 integrates an enhanced confidence module, including the key metrics such as the predicted local distance difference test (pLDDT), predicted aligned error (PAE), and the newly introduced predicted distance error (PDE). pLDDT values range from 0 to 100, with scores above 90 indicating very high confidence [43]. Lower PAE values reflect well-defined relative positions and orientations between residue pairs [35], while lower PDE values indicate higher accuracy of predicted inter-residue distances [21].

### Advantages

Compared with conventional structure prediction approaches, AF provides unprecedented structural coverage for biological research. The AlphaFold DB, based on AF2, provides extensive structural coverage of the UniProt proteome, encompassing the human proteome and 47 additional organisms. By predicting structures for over 200 million protein sequences, it offers researchers large-scale and freely accessible protein structure resources [44]. This extensive coverage helps bridge the long-standing sequence–structure gap, as only approximately 251,000 experimentally determined protein and peptide structures were available in the PDB despite the existence of over 203 million sequences in UniProtKB as of January 2026.

Beyond its extensive structural coverage, AF exhibits significant potential in protein structure prediction, multi-molecular complex prediction, and virtual screening, with accuracy far exceeding that of homology modeling and traditional docking methods. Unlike homology modeling, which relies on the availability of structurally resolved homologs, AF2 leverages a neural network-based architecture to achieve accurate predictions even in the absence of suitable templates [45]. This confers strong generalization capability, particularly for challenging targets such as transmembrane proteins, which are intractable to traditional modeling approaches [46, 47]. For example, benchmark assessments have shown that AF2 models outperformed homology models generated by SWISS-MODEL [48] and MODELLER [49]. In a prospective study targeting TAAR1 (a G protein-coupled receptor without an experimental structure), virtual screening of over 16 million compounds achieved a hit rate of 60% using AF2-predicted structures, compared with only 22% for homology models following experimental validation of top-ranked candidates [50]. Similarly, molecular docking screens for the 5-HT2A receptor achieved comparable success rates using AF2 models (26%) and cryo-electron microscopy (cryo-EM) structures (23%), demonstrating that AF2 predictions can approach experimental accuracy [51]. These findings confirm that AF2-predicted structures not only outperform homology models but, in some cases, rival experimentally determined structures in virtual screening applications.

AF2 provides effective solutions for the lack of suitable homologous templates in NP applications by enabling accurate modeling of diverse targets. Furthermore, the high-accuracy models of AF2 achieve higher hit rates in virtual screening than traditional homology models—even matching the performance of experimentally determined structures in certain cases. The following sections detail the specific applications and prospects of the AF series in NP research.

## Research progress on AlphaFold-derived models

AF-derived models alleviate critical limitations of static structure prediction through four complementary directions. For conformational dynamics, AlphaFolding [52], DynamicBind [53], and AlphaFold-Metainference [54] capture protein motions that rigid models poorly represent. For scalability, ColabFold [55] enables high-throughput batch prediction. For integrative prediction, SPOC [56] incorporates both structural and biological features to improve predictive performance. For specialized targets, FragFold [57], OpenFold [58], DiffBindFR [59], and AlphaFold with a cyclic offset [60] enhance accuracy for specific target classes. A comparative summary of commonly used AF-derived models is provided in Table 2.

**Table 2:** Comparison of characteristics, application, advantages, and limitations of nine common AF-derived models

Model	Characteristics	Application scenarios	Advantages	Limitations	References
AlphaFolding	A unified diffusion model capable of generating dynamic protein structures; generates full backbone and side-chain structures	Predicts dynamic protein structures across multiple time steps	Simultaneous trajectory prediction; captures conformational changes; strong temporal structural coherence	High computational demand for dynamic predictions; training relies on large-scale MD datasets	[52]
DynamicBind	Dynamic docking; handles conformational changes across picosecond to millisecond timescales	Reveals drug targets not identifiable in static structures	Efficient sampling; high-precision prediction	Dependence on apo-like structures from AF	[53]
AlphaFold-Metainference	Integrates AF-predicted inter-residue distances with MD simulations; uses the maximum entropy principle for structural constraints	Predicts conformational ensemble of ordered and disordered proteins	Prediction of inter-residue contacts in disordered proteins without specialized training	Limited range for distance prediction	[54]
ColadFold	Combination of MMseqs2-based homology search and AF2/RoseTTAFold; Python library and Jupyter notebook	Large-scale batch prediction of protein structures	Open-source; faster prediction speed than AF2 without sacrificing accuracy	Validation of prediction accuracy for orphan proteins is necessary	[55]
SPOC	Integrates structural and biological features; mitigates false positives for AFM	Rapidly filters high-confidence PPIs from the large-scale AFM screens	Strong screening and enrichment capability	Risk of false negative omissions; requirement for experimental validation	[56]
FragFold	Weighs the number of binding contacts by ipTM	Discovers inhibitory peptides for target proteins	High prediction efficiency; predicts unknown binding modes	Relies on AF; static binding modes only; fragment length limits	[57]
OpenFold	Staggered and multi-scale learning of secondary structure	Predicts long proteins, multimers, and protein–ligand complexes	Superior computational efficiency; less data-dependent; open-source	Gradient clipping; training instability; distillation template error	[58]
DiffBindFR	A full-atom diffusion-based flexible docking model; optimizes ligand overall movement, flexibility, and binding pocket side-chain torsion	simulation of protein–ligand binding structures accurately	Full binding pocket optimization; superior to traditional flexible docking methods; conforms to physical plausibility	Reduced accuracy for cryptic sites; increased PoseBusters penalties due to ligand–protein clashes	[59]
AlphaFold with a cyclic offset	Develops a cyclic offset; only modifies the cyclic peptide region; leaves the protein unchanged	Prediction of cyclic peptide–protein interactions; design of de novo cyclic peptides	Higher accuracy than classical docking tools	Risk of hallucination issues; not validated on larger datasets	[60]

## Structural prediction of canonical targets of natural products

**Fig. 4:**
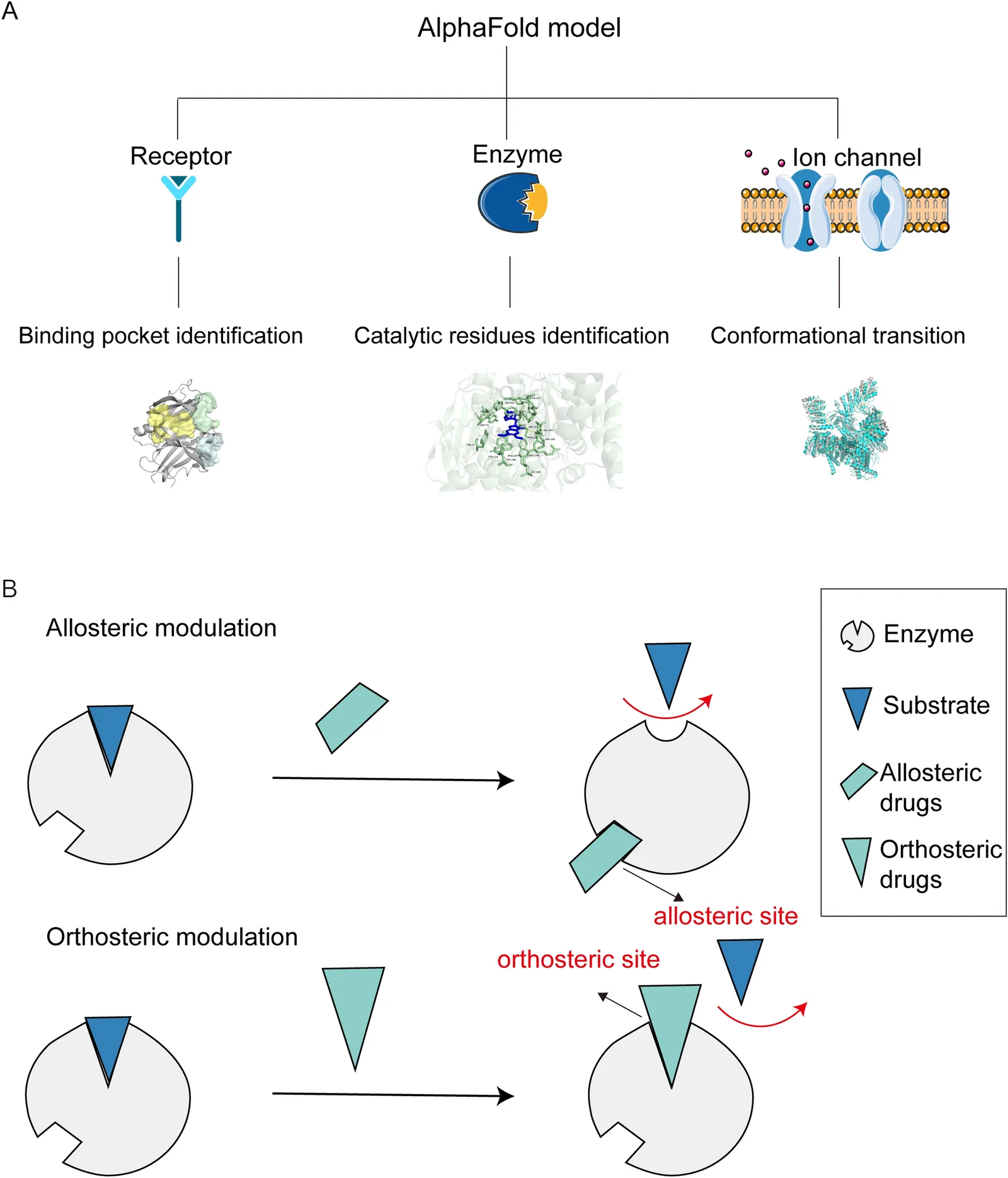
AF-based structures of canonical targets and allosteric/orthosteric regulatory mechanisms. **A** AF models of receptors, enzymes, and ion channels enable binding pocket identification, catalytic residue identification, and conformational transition analysis, respectively. **B** Schematic illustration of allosteric and orthosteric modulation using an enzyme as an example

NPs are a vital source of drug discovery, but the scarcity of structural information for NP targets remains a major obstacle to their development [9]. In structure-based drug design (SBDD), protein structures are primarily obtained from databases such as the PDB, with a limited number and diversity of available structures, rendering existing databases inadequate for NP research [61]. Major classes of drug targets—including receptors, enzymes, and ion channels—often contain allosteric sites that modulate activity beyond the orthosteric site (Fig. 4).

### Receptors

AF helps bridge structural gaps in signal transduction-related proteins, providing accurate structural models and binding site information for NP virtual screening.

For human disease-related targets, AF modeling compensates for missing structural data when PDB entries are unavailable. In a mechanistic study of *Lonicerae Japonicae Flos* as an adjuvant therapy for non-small cell lung cancer, AF2 was used to model potential targets PECAM1 and SPP1, supplying missing structures and providing reliable templates for docking analyses [62].

AF also demonstrates value in cross-species target prediction. Khan et al. [63] used vaccinia virus TMPK as a template to model monkeypox virus TMPK by AF2. Abdelkader et al. [64] employed AF3 to model NSP6, a SARS-CoV-2 protein lacking experimental structural data, and subsequently identified the potential NP-binding sites using CASTp and PrankWeb.

These studies highlight the utility of AF in reconstructing missing receptor structures and enabling downstream functional analyses.

### Enzymes

Many NPs exert biological activity by targeting catalytic proteins through orthosteric or allosteric mechanisms, underscoring the value of AF in structure-based investigations of metabolic transformations.

In orthosteric regulation, AF provides high-accuracy three-dimensional (3D) structures for target enzymes—particularly those lacking experimental structures or with low sequence identity to known templates. In the investigation of the traditional Chinese herbal formula (Qigui Jiangzhi Formula) for metabolic-associated fatty liver disease, AF3 predicted the structures of key enzymes, including AMPK and SIRT1. These models, combined with experimentally identified constituents, were used for molecular docking and molecular dynamics simulations for active component screening [65]. Moreover, AF enables subtype-level analyses, supporting the identification of natural bioactive compounds with improved selectivity. AF3 successfully modeled human tyrosinase, overcoming the limitations of homology-based approaches for targets with low sequence identity [66]. Comparative analyses of AF2-predicted homologous protein structures further guided the design of selective compounds, reducing potential off-target effects and toxicity [67].

In allosteric regulation, research focuses on conformational dynamics and domain organization. AF has been used to analyze conserved and flexible motifs, facilitating the detection of allosteric sites. Since the available crystal structure of taxadiene synthase represents an inactive open conformation, AF2 was employed to predict the catalytically competent closed state. This prediction revealed how N-terminal residues and three loops cooperatively seal the active site, enabling discrimination between the distinct allosteric roles of residues R578 and R580, which are inaccessible in the inactive crystal structure [68]. What’s more, hydrogen–deuterium exchange mass spectrometry (HDX-MS) provides experimental insights into protein conformational flexibility and dynamic regions, which can be spatially mapped onto the AF2-predicted structure of Class II lanthipeptide synthetases. This integration of HDX-MS data with AF2-derived models further enables the identification of conserved or variable domains involved in conformational transitions, offering a mechanistic framework for interpreting NP-induced allosteric effects at the molecular level [69].

### Ion channels

AF has emerged as a valuable structural tool for investigating NP interactions with ion channels, particularly in cases where experimental structures are incomplete or unavailable. AF has been integrated into ion channel drug discovery workflows to facilitate structure-based analysis of channel-ligand interactions and subtype selectivity [70].

In studies of peptide toxins from natural sources, AF3 models complement experimentally determined structures by reconstructing functionally relevant channel conformations. The centipede toxin SsTx-4 acts as a pore blocker on inwardly rectifying potassium channels. AF3-assisted structural analysis elucidated the pore-blocking mechanism by resolving channel regions critical for toxin recognition, which were not fully captured in experimental structures [71].

In summary, these studies demonstrate that AF enables structural characterization of diverse NP targets, including receptors, enzymes, and ion channels, and provides excellent structural support for analysis of ligand binding, conformational dynamics, and mechanistic exploration, thereby accelerating NP-based drug discovery.

## Hit-to-lead discovery and optimization

### AlphaFold-enabled hit-to-lead discovery and optimization: a structure-based virtual screening case study

**Fig. 5:**
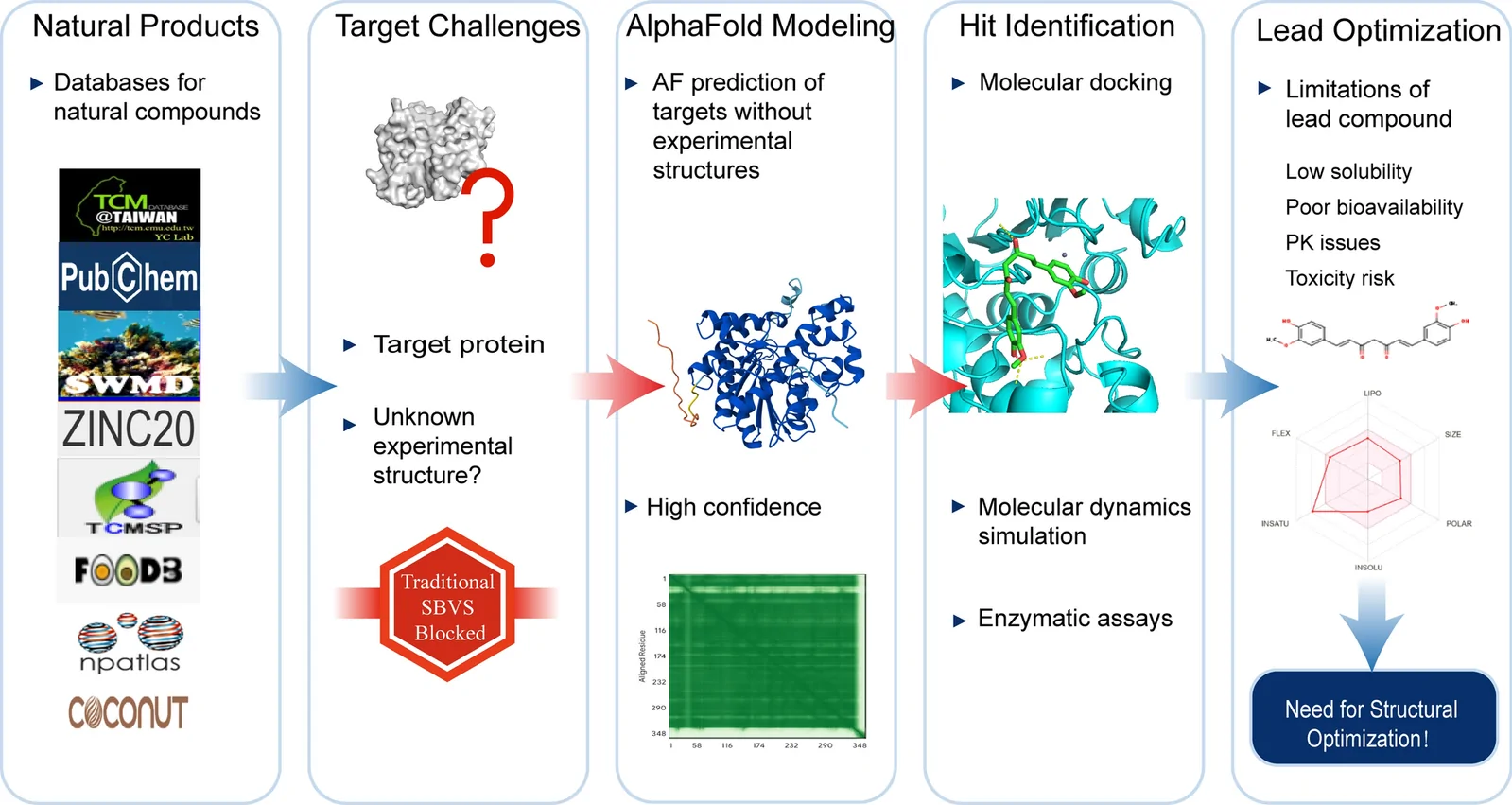
AF-enabled workflow for hit identification and lead optimization of NPs. AF-predicted target structures address the structural limitations of traditional structure-based virtual screening (SBVS) and enable molecular docking, molecular dynamics simulations, and experimental validation for hit identification, followed by structure-guided lead optimization to enhance drug developability

NPs remain a vital source of lead compounds [72]. However, their clinical translation is often limited by poor aqueous solubility, low oral bioavailability, suboptimal pharmacokinetic properties, and potential toxicity, as exemplified by curcumin, rhein, silibinin, and triptolide [73–76]. Structure optimization guided by the interaction modes between lead compounds and their target proteins is an effective strategy to overcome these limitations [77]. When the 3D structures of target proteins are available, SBDD becomes feasible. By enabling atomic-level prediction of protein structures, AF offers a powerful and efficient tool for structure-guided optimization of NP-derived leads [78]. And lead compound optimization generally follows a systematic workflow: target structure determination, pharmacophore extraction, molecular docking coupled with molecular dynamics simulations, in vitro and in vivo experimental validation of hit compounds, and subsequent lead structure optimization (Fig. 5).

Natural histone deacetylase (HDAC) inhibitors often possess complex molecular scaffolds, which can compromise poor stability and solubility, limiting their druggability [79]. Moreover, the absence of crystal structures for certain HDAC isoforms, such as HDAC11, has impeded the mechanistic understanding of natural inhibitor binding [80]. AF2-predicted models of HDAC11 were refined through incorporation and optimization of the Zn^2^⁺ ion, enabling accurate positioning of the metal center within the lysine-binding pocket, removal of steric clashes, and reproduction of the bidentate chelation mode observed in inhibitor binding [81]. Subsequent analyses revealed that the natural inhibitor trichostatin A (TSA) forms stable hydrogen bonds with His142, His143, and Tyr304 of HDAC11. Pharmacophore features derived from the high-confidence TSA–HDAC11 complex yielded 12,154 candidate compounds consistent with the binding mode. Further refinement through docking, molecular dynamics simulations, subtype-selective filtering, and enzymatic assays led to the identification of the hit compound ZINC000028464438 [82]. Based on known inhibitor binding modes, the core pharmacophoric features of HDAC11 inhibitors were defined, suggesting that active-site structure derived from AF2 models can guide core scaffold design and enable precise modulation of polarity, binding orientation, and substituent placement [83].

AF-predicted target structures provide a valuable framework for mapping key interactions (e.g., hydrogen bonds, salt bridges, metal coordination) between ligands and binding pockets, thereby facilitating the identification of essential pharmacophores. In addition, high-confidence models allow large-scale virtual screening of NP libraries, streamlining hit identification and lead optimization [84, 85]. By preserving key pharmacophores while introducing fragment replacement (e.g., removal of metabolically labile fragments) or polarity modifications (e.g., improvement of aqueous solubility), this approach establishes an efficient paradigm for developing NP-derived therapeutics.

### Hit identification via AlphaFold-based co-folding: a novel structure-guided virtual screening strategy

Co-folding is a deep learning framework that predicts protein structures and their interacting ligands in a synchronous manner [86]. AF3 has revolutionized the paradigm of protein–ligand prediction, achieving higher accuracy than conventional docking methods in many cases. Recent studies have demonstrated its applicability in both covalent screening and diverse ligand-binding scenarios, indicating its potential in virtual screening of small-molecule NPs.

A recent study established COValid, the first dedicated benchmark dataset for covalent protein–ligand screening. This dataset contains 874 active acrylamide-based protomers and 37,919 property-matched, topologically distinct decoys, spanning 10 cysteine attachment sites across 9 protein targets (including 8 kinases and KRAS G12C). Using this benchmark, AF3 achieved an average adjusted LocAUC of 71.8 ± 5.9%, markedly outperforming traditional docking methods such as DOCK6 (12.1 ± 9.9%). The performance of AF3 ranged from 56.4% (BMX) to 83.8% (FGFR4), approaching the theoretical maximum of 85.5%. Importantly, ablation experiments excluding highly similar actives confirmed the robustness of these results. Moreover, AF3 retains strong predictive capability in noncovalent binding scenarios, indicating that its applicability is not constrained by the type of interaction [87].

Further evaluations were extended to both covalent and non-covalent protein–ligand interactions. AF3-like models—especially those employing min-iPAE-based confidence scores (including AF3 min-iPAE and Boltz-2 min-iPAE)—performed stably across key evaluation metrics (AUROC, BEDROC, enrichment factors [EF]) on datasets such as DEKOIS2.0. These models achieved mean AUROC of 0.913, mean BEDROC of 0.755, and mean EF_0.5%_ of 22.98, outperforming traditional docking tools (e.g., Glide, AutoDock Vina) and some artificial intelligence (AI)-based docking tools (e.g., Gnina) [88]. Notably, AF3-based strategies maintained superior predictive accuracy even under challenging conditions characterized by limited training data and low ligand similarity.

Collectively, these studies highlight the emerging potential of AF3-based co-folding for structure-guided virtual screening. Its strong performance largely stems from the discriminative power of confidence metrics such as ipTM and min-iPAE, which effectively distinguish active compounds from decoys. Importantly, these metrics exhibit stable behavior across both covalent and noncovalent binding scenarios. As a result, AF3-based strategies demonstrate notable potential for early enrichment and prioritization of bioactive compounds, providing a promising framework for hit identification in NP-oriented drug discovery.

## Structural prediction and interaction analysis of macromolecular natural products with their targets

Macromolecular NPs—including proteins and peptides—present significant challenges for structural and interaction analyses. Unlike conventional docking approaches that rely on predefined protein structures, AF-based co-folding enables the simultaneous prediction of target proteins and their interacting partners, thereby facilitating inference of drug–target binding modes through coordinated folding and interface formation [89, 90]. Based on this framework, we further explore the specific application of AF in different types of multimolecular complexes.

### Peptide natural products

PNPs constitute an important source of drug discovery, characterized by high specificity and diverse bioactivities. They are widely distributed across plants, animals, and microorganisms. Approved pharmaceuticals derived from natural peptides include the antibiotic vancomycin, the anticancer agents actinomycin D and romidepsin, the analgesic ziconotide, and the anthelmintic emodepside [91–93]. PNPs exhibit pharmacological effects spanning antioxidant, anticancer, antibacterial, anti-inflammatory, antidiabetic, antihypertensive, and immunomodulatory activities, offering promising intervention strategies for a broad range of major diseases [94–99].

PNPs display two prominent structural characteristics: short sequences and substantial diversity in both sequence composition and 3D conformations, accompanied by more complex interaction interfaces than those of small-molecule ligands [100]. These features have resulted in a scarcity of experimentally resolved peptide structures, with critical conformational information often remaining unexplored. Consequently, traditional structure-dependent computational approaches show restricted applicability in PNP research [101, 102].

#### Structural prediction of peptide natural products

With the emergence of AF and related structural prediction technologies, research has increasingly focused on peptide-based NPs [103]. From 2016 to 2020, 3,975 research papers on bioactive natural peptides were published in PubMed, while this number increased to 6271 from 2021 to 2025, representing a 57.8% surge over the five years. Publication growth has accelerated over the past three years, highlighting PNPs as an emerging research hotspot. This rapid expansion has, in turn, underscored the growing demand for efficient and accurate structure prediction approaches, motivating the application of AI-based models such as AF.

AF demonstrates distinct advantages in modeling peptides of varying lengths through flexible prediction strategies. Especially for peptides shorter than 30 residues, AF achieves accuracy unattainable by conventional homology modeling [102]. Liu et al. employed AF2 to predict the structures of three anticancer peptides (ACPs) from *Sanghuangporus sanghuang*. For the long-chain ACP comprising 31 amino acid residues, direct prediction revealed a predominant α-helical secondary structure. In contrast, for the hexapeptide, the corresponding full-length precursor protein was first identified from databases, its structure was predicted, and the active peptide segment was subsequently extracted, yielding an accurate structural model for downstream molecular docking [104].

Beyond its adaptability to peptide length, AF demonstrates a strong capability for analyzing structurally diverse peptide conformations, which has long challenged the structure elucidation of PNPs. For example, AF3 was applied to analyze 36 ShK domain peptides from four species of sea anemone, identifying structural determinants that govern binding stability and specificity, and yielding molecular-level insights into structure–function relationships [105]. Furthermore, large-scale peptide structure predictions generated by AF2 facilitate the construction of structural databases, laying the foundation for the acquisition of structural data and the discovery of novel bioactive peptides. Representative applications include AF2-based modeling of uncharacterized antiviral peptides to expand existing databases [106]. And the use of AF2-predicted antimicrobial peptide structures as benchmark datasets to train the deep AMPNet model achieves high accuracy in antimicrobial peptide identification. Importantly, these predicted structures have been validated for downstream functional analysis [107, 108].

Overall, AF accommodates peptides across varying sequence lengths and structural conformations, providing a foundation for PNP research. The structure-based databases and predictive models derived from AF-predicted structures have expanded the scope of PNP screening and mechanistic research, driving increased scientific interest in this field [109].

#### Interaction analysis between target proteins and peptide natural products

Despite the abundance of bioactive peptides in NPs, a major bottleneck in PNP research arises from their flexibility and conformational heterogeneity, which result in binding modes that differ significantly from those of small molecules, making accurate prediction via conventional molecular docking challenging. AF-based methods enable accurate prediction of the binding modes and key sites in peptide-receptor complexes without relying on templates [17, 36, 55, 110–112]. Therefore, the AF-based approaches are expected to accelerate and improve the accuracy of peptide discovery [113].

The high accuracy of AF in predicting PPIs has also enabled mechanistic studies in sensory biology and the rational design of food flavor profiles. Yan et al. applied AF3 to model complexes between the umami receptor T1R1 and 44 candidate peptides identified from two tea cultivars, Longjing 43 (LJ43) and Qunti (QT). Their analysis identified peptides with potential binding affinity to T1R1, clarifying the potential umami recognition cores [114].

Similarly, in a study investigating bitter peptides derived from casein degradation, Richter et al. [115] used AFM to model the complex between the polypeptide VAPFPEVF and the TAS2R16 receptor, and subsequent molecular dynamics simulations revealed that the peptide occupies the same binding site as salicin, providing a structural basis for competitive inhibition and receptor modulation.

### Proteinaceous natural products

#### Structural prediction of proteinaceous natural products

Protein-based NPs refer to bioactive macromolecules derived from natural sources, including venom toxins, enzyme-type proteins, and plant-derived bioactive proteins, serving as important resources for drug discovery and chemical biology research due to their distinctive 3D architectures and potent bioactivities [116–118]. Animal and marine venoms are frequently explored as therapeutic agents due to their potent bioactivity in targeting ion channels, as exemplified by representative components summarized in Table 3.

**Table 3:** Overview of newly identified structures of natural product-source proteins by AF

Source	Bioactive protein	Pharmacological activities	Uniprot ID	Structure	References
Scorpion	Phospholipase A_2_	Acts as adjuvants in anticancer therapy	P0DKU2		[99]
	Hyaluronidase	Improves targeted drug delivery	P86100		[99]
Helix pomatia	Hemocyanin	Acts as an immunoadjuvant and immunomodulator	P80960		[158]
Mastigocladus laminosus	Phycobiliprotein	Exerts antioxidant, antibacterial, and antitumour activities	P07553		[159]
Snake	Contortrostatin	Exhibits anti-angiogenic and anti-cancer effects; acts as an adjuvant in chemotherapy for melanoma and viral infections	Q9IAB0		[160]
Leech	Hirudin	Exerts anticoagulant activity	P28504		[161]
Earthworm	Lumbrokinase	Acts as an adjunctive therapy for acute ischemic stroke	Q25394		[162]

Nevertheless, a large proportion of proteinaceous NPs remains unexplored. Many originate from less-studied organisms and lack experimental structures, which impedes molecular mechanistic investigations of their pharmacological effects [119]. It is known that snake venom contains 19,000–25,000 toxins, but only 2000 of them have been described, with less than 10% having experimental structures [120].

Deep learning-based structure prediction methods, particularly AF, have accelerated the discovery of proteinaceous NPs. By generating high-accuracy structural models, AF enables the structural characterization of proteins that are otherwise experimentally inaccessible. For instance, Kalogeropoulos et al. used AF2 to predict over 1000 snake venom protein models lacking experimental data, with most predictions falling within the confidence and high-confidence ranges [120].

#### Interaction analysis between proteinaceous natural products and target proteins

PPIs mediate the recognition between proteinaceous NPs and their biological targets [121, 122]. AF2 and AFM have achieved breakthroughs in predicting protein complex structures. A review by Tsuchiya et al. noted that AFM has been successfully applied to predict heterologous PPIs, outperforming traditional docking-based methods such as ZDOCK [123]. This advantage is particularly evident in challenging systems such as antibody–antigen complexes, where AFM outperforms RoseTTAFold and other methods [124]. Furthermore, through large-scale sampling, the medium-to-high accuracy rate of AFM in CAPRI has been significantly improved to over 50% [125, 126].

The present studies have primarily focused on PPIs involved in physiological and pathological processes, whereas systematic benchmarking and case studies of interactions between proteinaceous NPs and targets remain scarce. Nevertheless, from a mechanistic perspective, interactions between exogenous proteinaceous NPs and endogenous human targets are as complex as antibody–antigen recognition. AFM shows capabilities in modeling non-coevolved protein systems and predicting the interface accurately through extensive sampling. Extending these capabilities to target identification and mechanistic elucidation of proteinaceous NPs represents a promising and largely untapped research direction.

## AlphaFold-enabled strategies for natural product molecular glues targeting protein–protein interactions

### Druggability challenges of PPI interfaces

**Fig. 6:**
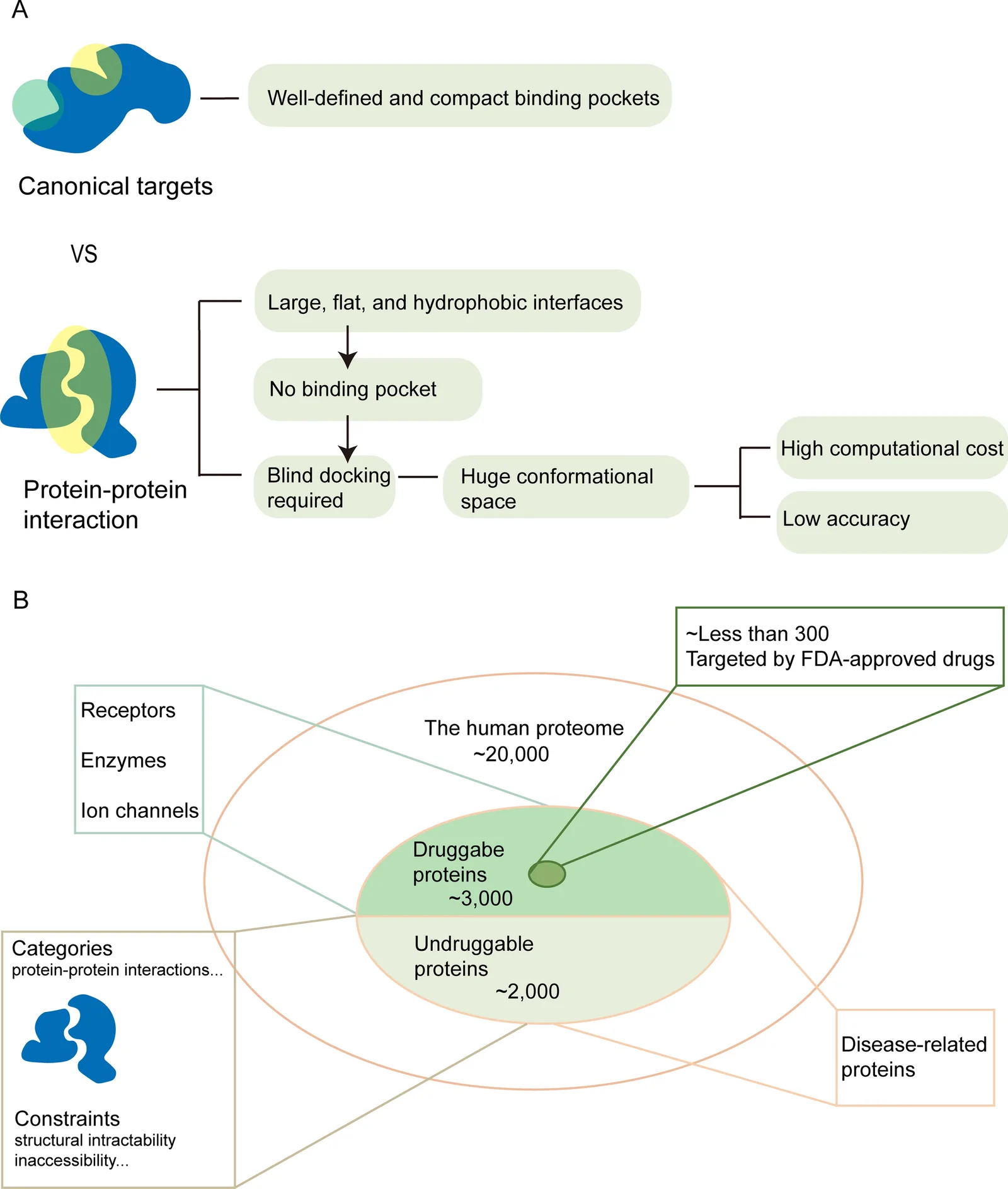
Undruggable factors of human targets and analysis of current druggable and undruggable targets. **A** Factors underlying the perception of protein–protein interactions (PPIs) as undruggable compared to classical targets. **B** Only approximately 1.5% of human proteins are targeted by FDA-approved drugs, while many remain undruggable, including targets involved in PPIs. Data sources: Illuminating the Druggable Genome (IDG) and Online Mendelian Inheritance in Man (OMIM)

Unlike classical drug targets with well-defined, compact binding pockets, PPIs typically occur across large, relatively flat, and hydrophobic interfaces, which have long been regarded as “undruggable” and difficult to modulate by conventional drug design strategies [127] (Fig. 6A). This inherent undruggability of PPIs means that a vast majority of the human proteome remains untargetable, as depicted in Fig. 6B. However, certain NPs act as MGs, inducing the formation of protein–NP–protein ternary complexes [128–132]. MGs have emerged as a promising strategy for modulating PPIs, either by stabilizing endogenous protein interactions or by inducing de novo PPIs through cooperative binding at the interface to form stable ternary complexes [133]. For example, the NPs fusicoccin A, cotylenin A, and their semisynthetic derivatives act as MGs to stabilize the PPI interface between the hub protein 14-3-3 and its client proteins [134].

Despite these advances, major challenges remain in elucidating the binding modes of NP-mediated PPIs and the associated conformational changes in interacting proteins.

### AlphaFold as a structural solution for natural molecular glue research

AF3 and AFM provide a structural framework for modeling protein complexes and analyzing PPI interfaces as tools for the technical challenges in natural product MG research [135–138]. In the absence of experimental structures, AF3-generated models can serve as reliable starting configurations for molecular dynamics simulations [139, 140]. Furthermore, the co-folding strategy of AF3 allows direct prediction of ternary complexes [141]. Confidence metrics—including pLDDT and PAE—further enable quantitative assessment of interface quality and structural reliability [21]. In practical applications, prediction reliability can be improved by focusing on high-confidence regions and considering the functional structural state, such as removing non-interacting regions that may introduce steric constraints or mask interaction motifs [142].

Nevertheless, the direct application of these approaches to NP-mediated PPI modulation remains limited. AF-predicted structures often resemble apo-like conformations, whereas docking-based approaches typically rely on holo-like binding sites, potentially leading to inaccuracies in modeling ligand-induced interactions [143]. On the other hand, weak or transient PPIs with limited co-evolutionary signals and poorly defined interfaces remain challenging to predict. For example, AFM relies on co-evolutionary signals derived from MSA, while AF3 co-folding approaches also utilize evolutionary information. However, MG-induced PPIs are frequently weak or even non-interacting in their native states and therefore lack strong evolutionary constraints [144, 145]. Finally, current models are not specifically trained on MG-mediated ternary complexes, which limits their generalizability to these systems [146]. Both docking-based and co-folding-based strategies are subject to these issues, highlighting the need for methodological refinement.

### AlphaFold-based strategies for characterizing molecular glue-mediated ternary complexes

**Fig. 7:**
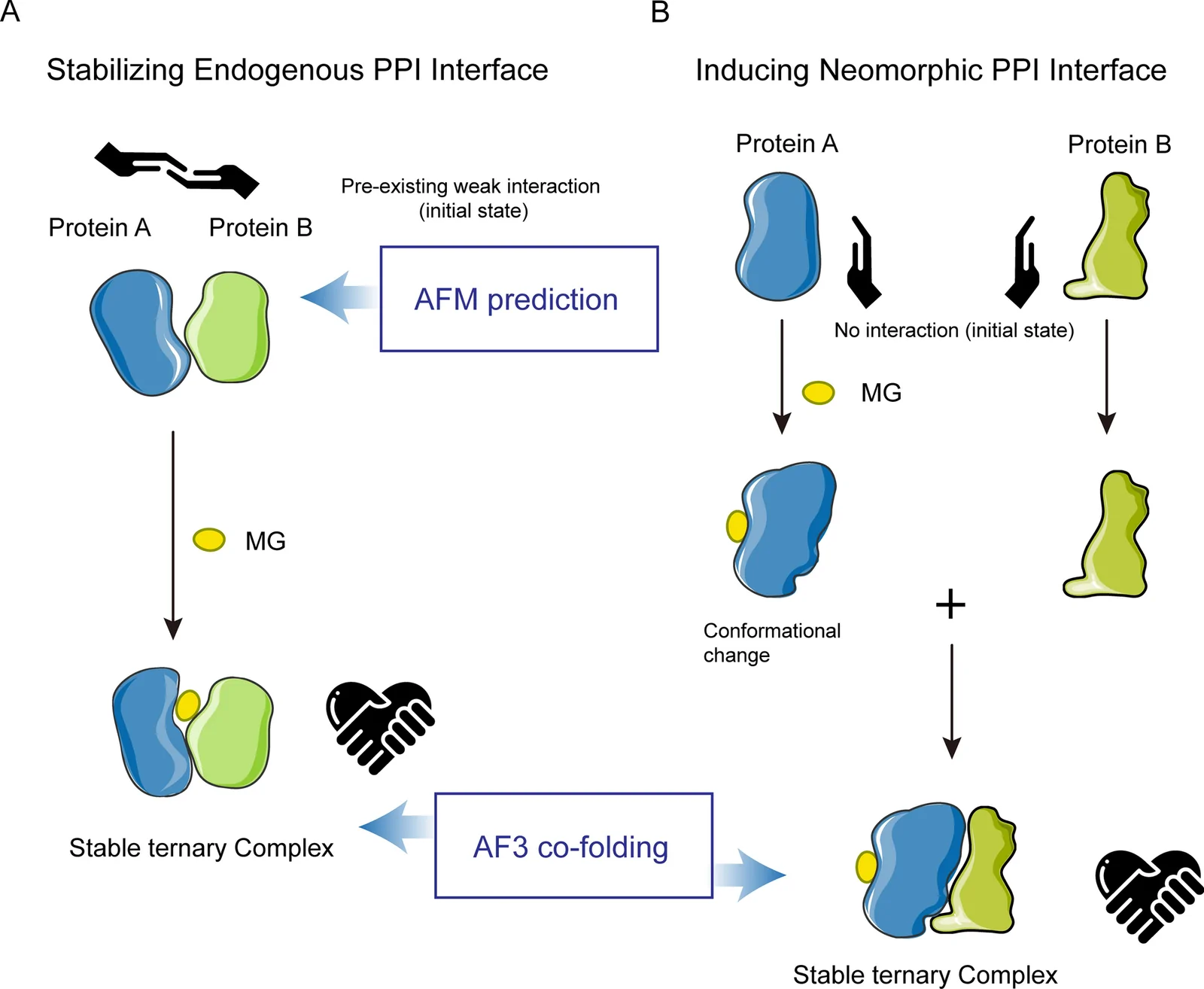
Typical applications of AlphaFold-Multimer (AFM) and AF3 co-folding in modeling molecular glue (MG)-mediated PPIs. **A** Pre-existing weak interactions modeled by AFM or AF3 co-folding. **B** Initially non-interacting proteins modeled by AF3 co-folding to directly predict MG-induced ternary complexes with conformational changes and de novo PPI formation

Two complementary computational strategies have been developed for the structural characterization of MG-mediated ternary complexes. The first combines AFM for protein complex prediction with molecular docking and dynamics simulations to characterize interaction modes (Fig. 7A). The second exploits the co-folding capabilities of AF3 to directly predict protein–ligand–protein ternary assemblies (Fig. 7B).

#### AlphaFold-Multimer-based prediction coupled with molecular docking

A recent study systematically investigated two pathways of MG-mediated complex formation by AFM and docking protocols [147]. In the first pathway—where the protein–protein interface pre-exists before ligand binding, AFM was used to predict the binary complex, followed by docking of the ligand at the interface. This strategy was exemplified in the GluN1b–ifenprodil–GluN2B system, where ifenprodil and 93 analogs were docked onto the AFM-predicted interface. In the second pathway, the small molecule initially binds to one protein and subsequently recruits a partner protein. Because the two proteins are originally non-interacting, direct AFM modeling of protein pairs was often unsuitable for these MG systems, such as CRBN–pomalidomide–IKZF1 and FKBP–rapamycin–FRB. In these cases, a stepwise strategy combining initial ligand docking with subsequent incorporation of the second protein was employed, yielding ternary complexes with high structural agreement.

#### Direct ternary complex prediction via AlphaFold3 co-folding

In contrast to sequential approaches, AF3 co-folding predicts ternary complexes directly by integrating the SMILES strings of natural MGs with the amino acid sequences of the protein partners, with a specific focus on analyzing how MGs induce de novo PPI formation or stabilize native interactions [146]. The utility of this approach is further exemplified by the development of a comprehensive database of protein–MG–protein ternary complexes, which integrates two types of ternary complex structures: 317 experimentally determined structures directly collected from the PDB, and additional complexes generated by AF3. For the AF3-generated models, only predictions with MG-chain ipTM above 0.68 and average pLDDT above 70 were retained. This filtering strategy achieved an F1 score of 0.71, with a precision of 0.58 and a recall of 0.93, yielding 3607 high-confidence ternary complex models. The database provides comprehensive resources, including 3D structure visualization, 2D protein–MG interaction maps, prediction metrics, ternary complex annotations, experimental information, and associated references [148].

## Challenges, complementary solutions, and emerging advances in AlphaFold-based structure prediction

AF has demonstrated potential in NP research; however, several challenges must be carefully considered to ensure its reliable application [22]. Concurrently, the rapid advancement of AI-driven structural biology continues to generate next-generation platforms that may overcome existing limitations. This section discusses current challenges and mitigation strategies, followed by an overview of emerging technologies that extend beyond the framework of AF.

### Current limitations and complementary solutions

AF-predicted structures may exhibit stereochemical violations, including atomic clashes, peptide plane distortions, and chiral inversions. These issues can be mitigated by enforcing peptide bond geometry via violation-based loss terms, followed by structural relaxation via gradient descent in Amber, while preserving the global backbone fold [34, 149].

Another limitation lies in hallucinatory effects, particularly in the modeling of intrinsically disordered regions, where AF3 may incorrectly predict compact and ordered conformations [150, 151]. To alleviate this limitation, a cross-distillation strategy has been proposed, in which structures predicted by AFM (v2.3) are incorporated into the training dataset [21, 152]. Because AFM tends to represent disordered regions as extended conformations, this approach improves the assignment of low-confidence regions as disorder or flexible loops, thereby reducing hallucination effects [150, 153].

Additionally, AF does not account for molecular dynamics and conformational flexibility explicitly [21], both of which are often critical for elucidating NP–target interactions. This is exemplified in the modeling of the human T-type voltage-gated calcium channel, where AF3 successfully complemented missing regions from experimental structures, but preferentially adopted an inactive conformation, limiting its docking with agonists. This behavior likely reflects a bias in AF3 toward predicting thermodynamically stable, ligand-free inactive conformations of ion channels [154]. Several approaches have been developed to incorporate dynamic information. Models like AlphaFolding, DynamicBind, and AlphaFold-Metainference integrate molecular dynamics simulation data to model conformational ensembles [52–54]. Complementarily, slow feature analysis—trained on short and unbiased molecular dynamics simulations initiated from AF2-generated conformational ensembles—enables the identification of rare but functionally relevant events, including cryptic pocket opening, protein–ligand binding, and allosteric regulation in kinases [155].

These challenges and corresponding mitigation strategies underscore critical considerations for applying AF-based models in NP research, particularly for systems characterized by structural flexibility, intrinsic disorder, or dynamic binding processes.

### Emerging advances: beyond AlphaFold3

While these mitigation strategies enhance the applicability of AF3, the field is rapidly progressing toward integrated platforms that unify structural prediction with pharmacological evaluation. A representative advance is the Drug Design Engine (IsoDDE), introduced by Isomorphic Labs in February 2026.

IsoDDE integrates structural modeling, binding affinity estimation, and cryptic binding site identification within a single system, directly circumventing limitations in AF3-based workflows. In antigen–antibody complex modeling, IsoDDE achieved substantially higher success rates than AF3. Additionally, the platform can infer cryptic binding sites directly from primary amino acid sequences, enabling rapid identification of active pockets [156].

Described as an “AlphaFold 4-level” advancement [157], IsoDDE has emerged as an advanced platform in structure-based drug discovery, bridging the gap between structural prediction and therapeutic candidate identification.

## Conclusion

Accurate prediction of protein three-dimensional structures from amino acid sequences remains a central challenge in structural biology. Conventional modeling and docking approaches are insufficient to bridge the structural data gap in natural product research—particularly for orphan proteins, poorly characterized targets, and flexible peptide conformations. AlphaFold, distinguished by its high generalization capability and well-calibrated confidence metrics, has pioneered an artificial intelligence-driven paradigm in structural biology, enabling precise NP screening, multimeric complex prediction, and binding mode analysis.

The large-scale application of AF facilitates high-throughput structure prediction and virtual screening with better accuracy, efficiency, and lower cost, accelerating the identification of bioactive NPs, elucidation of molecular mechanisms, and structure-based optimization. AF-derived structural insights contribute to a deeper understanding of pharmacological and toxicological processes, collectively promoting a transformative paradigm in NP-based drug discovery.

AF is expected to advance precision and personalized medicine through structure-based target identification and rational drug design, thereby expediting the clinical translation of NP-based therapeutics.

## Data Availability

Data availability is not applicable.
